# Phylogeny and Diversification Patterns among Vesicomyid Bivalves

**DOI:** 10.1371/journal.pone.0033359

**Published:** 2012-04-12

**Authors:** Carole Decker, Karine Olu, Regina L. Cunha, Sophie Arnaud-Haond

**Affiliations:** 1 IFREMER, REM/EEP/LEP, Laboratoire Environnement Profond, Plouzané, France; 2 CCMAR-CIMAR, Center for Marine Sciences, University of Algarve, Gambelas, Faro, Portugal; Biodiversity Insitute of Ontario - University of Guelph, Canada

## Abstract

Vesicomyid bivalves are among the most abundant and diverse symbiotic taxa in chemosynthetic-based ecosystems: more than 100 different vesicomyid species have been described so far. In the present study, we investigated the phylogenetic positioning of recently described vesicomyid species from the Gulf of Guinea and their western Atlantic and Pacific counterparts using mitochondrial DNA sequence data. The maximum-likelihood (ML) tree provided limited support for the recent taxonomic revision of vesicomyids based on morphological criteria; nevertheless, most of the newly sequenced specimens did not cluster with their morphological conspecifics. Moreover, the observed lack of geographic clustering suggests the occurrence of independent radiations followed by worldwide dispersal. Ancestral character state reconstruction showed a significant correlation between the characters “depth” and “habitat” and the reconstructed ML phylogeny suggesting possible recurrent events of ‘stepwise speciation’ from shallow to deep waters in different ocean basins. This is consistent with genus or species bathymetric segregation observed from recent taxonomic studies. Altogether, our results highlight the need for ongoing re-evaluation of the morphological characters used to identify vesicomyid bivalves.

## Introduction

Chemosynthetic ecosystems are found worldwide in the deep ocean and harbour specific communities that have a high level of endemism [1]. Some large families or genera, among which vesicomyid bivalves are one of the most remarkable examples, are distributed across a wide diversity of habitats including hydrothermal vents, cold seeps and whale carcasses. These inhabitants of the deep sea have developed intrinsic features that enable them to live in these extreme environments [2], [3], [4]. Similarly to many other taxa associated with chemosynthetic ecosystems, vesicomyids have chemoautotrophic bacteria in their gills that use sulphide components and provide their hosts with nutriments, thereby creating symbiotic relationships [5], [6], [7], [8]. Since the late 1970s and the discovery of chemosynthetic ecosystems, vesicomyid species have been described on the basis of morphological characters, and a taxonomical framework has been established. Within the currently described 112 extant and 4 fossil species, 47 have been described from cold seeps, 67 from putative seep environments and 14 from hydrothermal vents [9], [10], with some of these species sharing several environment types.

Initial phylogenetic studies have shown numerous discrepancies between the taxonomy based on morphological characters or on molecular data [11], [12], [13]. Phylogenetic relationships of Pacific and Atlantic vesicomyids reconstructed using the mitochondrial gene cytochrome oxidase subunit I (COI) supported the monophyly of this group [12], [13], [14], [15], [16], [17], [18], [19]. However, within the family Vesicomyidae, the morphologically defined genera *Calyptogena* and *Vesicomya* are not supported by molecular phylogenies which have revealed polyphyletic branching [12]. Moreover, the occurrence of cryptic species complexes (e.g. *C. pacifica*/*V. lepta*) and multiple trans-Pacific migrations suggest incomplete lineage sorting in an ongoing speciation process (e.g. *C. soyoae*/*C. kilmeri*) [12], [13], [16].

Recent revisions of the taxonomy of Vesicomyidae based on soft tissues and shell morphology include the description of several new species around the world [9], [10], [20], [21], [22], [23]. These studies advocate the separation of the family into two subfamilies (Vesicomyinae and Pliocardiinae), re-description of the *Calyptogena* genus and the creation of new genera separating species into distinct genera. The subfamily Vesicomyinae comprises only the genus Vesicomya (small-sized bivalves without reduced gut and subfilamental tissue on the gills) and the subfamily Pliocardiinae (medium and large body size with subfilamental tissue on the gills), which includes 15 genera. Consequently, most vesicomyids from hydrothermal vent and cold seep ecosystems initially attributed to *Calyptogena* and *Vesicomya* genera have been assigned to new erected genera (e.g. *Abyssogena*) or old ones (e.g. *Pliocardia*) [23], [24]. Nevertheless, the revision is under progress and some of these species are still assigned to *Calyptogena*. The Pliocardiinae are generally of large size, harbour sulphide-oxidizing bacteria and are highly specialized to sulphide-rich environments while symbiosis is not proved in small size Vesicomyinae not restricted to these environments [23], [24]. Both Vesicomyinae and Pliocardinae are distributed worldwide with a broad vertical range [24]. However, the geographic distribution and depth range vary among genera. Most genera (11) have colonised at least two oceans and only five have a strictly regional distribution. However, the vertical distribution of each genus is narrow except for two genera *(Vesicomya* and *Isorropodon*) that have colonised zones ranging from the sublittoral to abyssal. Recent revisions of vesicomyid genera demonstrate that genera generally occur in only one bathymetric zone [23], [24], [25].

In the present study, we investigated the phylogenetic relationships among recently described vesicomyid species in the Gulf of Guinea and their western Atlantic and Pacific counterparts using mitochondrial DNA COI sequence data. The reconstructed phylogeny was used to: (1) compare the taxonomic status of recently described species based on morphological characters with molecular based taxonomy and discuss possible discrepancies between both approaches, and (2) place these species in their phylogenetic context and perform an ancestral character state reconstruction to test previous hypotheses suggesting a relationship between vesicomyid diversification and the habitat/depth that they occur.

## Materials and Methods

### Specimens

**Figure 1:**
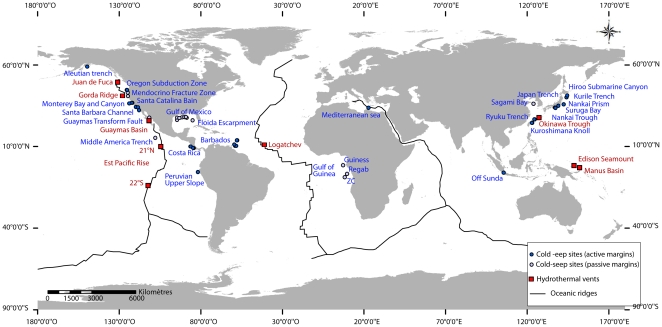
Localisation of sample sites indicated in table S1.

Several specimens of nine putative vesicomyid species were collected worldwide in cold seeps, including five in the Gulf of Guinea, and identified morphologically to the species or genus level and recently assigned to existing or new genus [9], [10], [25]: *Calyptogena valdiviae* Thiele and Jaeckel, 1931 [26], *Christineconcha regab* Cosel and Olu, 2009 [10] (here after *Ch. regab*), *Elenaconcha guiness* Cosel and Olu, 2009 [10], *Laubiericoncha chuni* Thiele and Jaeckel, 1931 [26], *Laubiericoncha myriamae* Cosel and Olu, 2008 [9], *Wareniconcha guineensis* (Thiele and Jaeckel, 1931), (Atlantic Ocean), *Calyptogena n. sp1*, *Calyptogena n sp2*, (Pacific Ocean) and *Isorropodon perplexum* Sturany, 1896 [27] (Mediterranean Sea) (Table 1, Figure 1). Sample collection was performed using deep submergence vehicles (Nautile during the DIAPISUB and NAUTIMATE cruises, the ROV Victor 6000 during the BIOZAIRE 2 cruise, and Quest 4000 (MARUM) during the RV Meteor expedition M70-3 [28] and during the expedition M76-3b GUINECO (MARUM) as well as by surface dredges (BIOZAIRE 3 cruise) (Table 1). In most cases, specimens were dissected after retrieval or frozen whole at −80°C. Species identification was performed by a taxonomist (R. Von Cosel) [10] or by the authors for new specimens of already described species from the Gulf of Guinea (KO, CD). Specimens unidentified to the species level and collected in the Middle America Trench off Mexico in the Pacific Ocean were named C. n sp (CI) and C. n sp (CII) (Tables 1 and S1).

**Table 1:** Location, cruise, water depth and number of specimens (N) sequenced.

Specimen	N	Location	Cruise	Latitude	Longitude	Water depth (m)	Dive number
*Christineconcha regab*	21	REGAB pockmark, site 1	GUINECO	S 5°47.84	E 9°42.62	3167	M76/3b_323, D211
*Christineconcha regab*	4	REGAB pockmark, site 2	GUINECO	S 5°47.87	E 9°42.69	3170	M76/3b_344, D217
*Christineconcha regab*	4	REGAB pockmark, site 3	GUINECO	S 5°47.98	E 9°42.48	3165	M76/3b_379, D225
*Laubiericoncha chuni*	3	REGAB pockmark, site 3	GUINECO	S 5°47.98	E 9°42.48	3165	M76/3b_323, D225
*Elenaconcha guiness*	1	Guiness pockmark	BIOZAIRE2	S 1°34.63	E 8°32.91	695	148
*Calyptogena valdiviae*	1	Guiness pockmark	BIOZAIRE2	S 1°34.57	E 8°31.81	687	148
*Wareniconcha guineensis*	1	ZC	BIOZAIRE3	S 7°42.41	E 10°00.84	4017	Chalut14
*Laubiericoncha myriamae*	1	Barbados Accretionary Prism, Orénoque B	DIAPISUB	N 10°16.38	W 58°35.67	2000	10
*Isorropodon perplexum*	2	Amsterdam Mud Volcano	M70-3	N 35°20.07	E 30°16.15	2025	M70/3 GeoB 11315, D134
*Calyptogena n. sp1*	1	Middle America Trench off Mexico	NAUTIMATE	N 18°21	W 104°21	3400	NM 09
*Calyptogena n. sp2*	1	Middle America Trench off Mexico	NAUTIMATE	N 18°21	W 104°21	3400	

### DNA extraction, amplification and sequencing

Total genomic DNA from the adductor muscles of 29 *Ch. regab*, three *L. chuni*, two *I. perplexum*, and one individual for each of the six remaining species, was isolated using the CTAB extraction method (Doyle and Doyle 1987). Purified nucleic acids were resuspended in DNase-free water and stored at −20°C. Universal (LCOI1490+HCOI2198 [29]) and vesicomyid-specific (VesLCO+VesHCO [12]) primers were used to amplify a partial fragment of the mitochondrial COI gene. PCR amplifications were carried out in 50 µl reactions with the following final concentrations: 1× Taq DNA polymerase buffer (Promega), 2.5 mM MgCl2, 0.20 mM dNTPs, 0.6 mM each primer, 0.5 U Taq DNA polymerase (Promega) and 5 µl of purified DNA. The following profile was used: incubation at 94°C for 90 s, 35 cycles of incubation at 94°C for 30 s, 45°C for 60 s, 72°C for 75 s and a final extension at 72°C for 10 min. PCR products (40) were then sent to GATC Biotech for sequencing in both directions. Accession numbers are given in Table S1.

### Molecular taxonomy and phylogenetic analyses

Mitochondrial COI gene sequences from all vesicomyid bivalves available in the GenBank database were included in all analyses. As Genbank contains data obtained prior to the new taxonomic revision, or even data from specimens either unnamed or later re-identified with a different species name, we renamed unidentified or misidentified specimens of sequences retrieved from GenBank according to their identification in the most recent taxonomic revisions by expert taxonomists [20], [23], [24]. For example *Calyptogena* sp. “Clam #2 [12] became *Abyssogena southwardae* following [23]. These new names given in table S1 are those used in the phylogenetic tree.

To assess the phylogenetic relationships of vesicomyids, we used 87 COI nucleotide sequences (including 12 sequences obtained in this study). Given their close relationship with vesicomyids, species belonging to families Arcticidae (A*rctica islandica*) Corbiculidae (*Corbicula fluminaris* and *Corbicula japonica*), Glauconomidae (*Glauconome chinensis*), Veneridae (*Venus antique*, *Anomalocardia brasiliana* and *Mercenaria mercenaria*) were chosen as outgroups [30]. Alignment of nucleotide sequences was conducted using Geneious Pro version 4.6 [31], and verified by eye to maximise positional homology. The Akaike's information criterion (AIC) [32] implemented in Modeltest v. 3.7 [33] was used to determine the evolutionary model that best fit the data set. PhyML v. 2.4.4 [34] was used to estimate the maximum likelihood (ML) tree using the model supported by Modeltest (GTR+I+G) model [35], and to test the robustness of the inferred trees using non-parametric bootstrap proportions (BPs) with 1000 pseudoreplicates. ML analyses were carried out through the freely available web-based portal Bioportal (http://www.bioportal.uio.no). Specimens provisionally assigned to existing genera or whose description is in progress following [24], are underlined by different colours.

**Figure 2:**
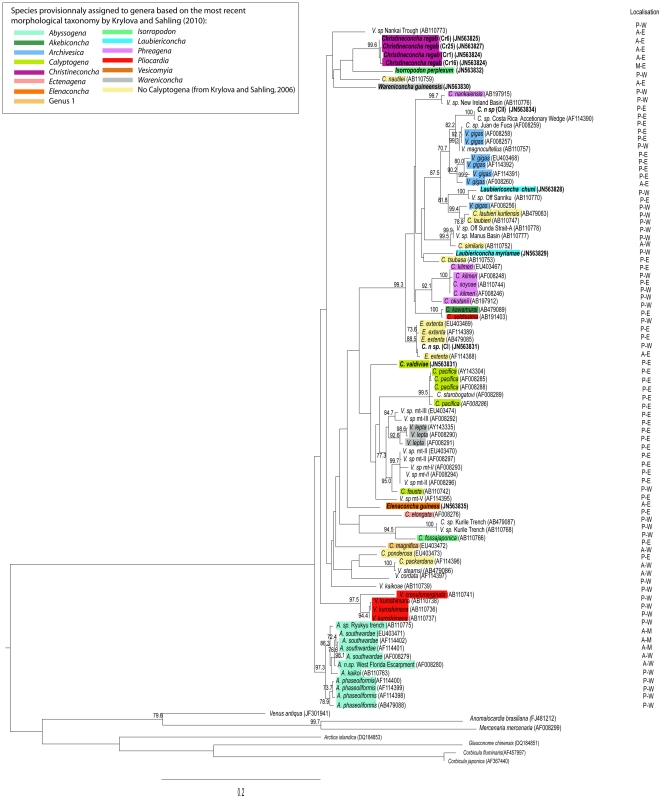
DNA maximum-likelihood tree based on COI nucleotide sequences rooted with *Glauconome chinensis*. The evolutionary model tested was GTR+I+G [35] (proportion of invariable sites = 0.39, number of substitution rates = 6, gamma distribution parameter = 0.54). Bootstrap values >75% for 1000 replicates are given above branches and clades. Specimens are named based on recent revisions. Colours correspond to genera from Krylova and Salhing (2010). Localisation: A, Atlantic Ocean; ME, Mediterranean Sea; P, Pacific Ocean and W, west; E, east; M, middle.

Ancestral character state reconstruction for the evolution of depth limits and habitat during vesicomyid evolution (53 species) was performed using MacClade v. 4.08 [36] to test whether the evolutionary pattern of habitat and depth was significantly correlated with the molecular phylogeny. The analyses based on the maximum depth limits of each taxon were performed using 13 depth intervals (see Figure 2 for further details) whereas ancestral reconstruction of the character “habitat” included four different categories (Seeps, Vents, Seeps and Vents, and Coastal). Each depth limit and different habitat was mapped onto the reconstructed ML topology. To obtain the null distribution of the number of transitions of the character “depth” and “habitat” 1000 random trees were generated. Tree lengths were compared with the mean number of steps of the null distribution of each reconstruction to test whether the observed number of transitions was significantly more correlated with the phylogeny than expected at random.

## Results and Discussion

### Molecular versus morphological taxonomy

The ML analysis based on a 630 bp fragment of the mitochondrial COI gene yielded the tree (−lnL = 5653.83) shown in Figure 2. Despite the rather low support of the deepest nodes, more recently diversified clusters of genera and species showed reasonable bootstrap support (>75%) on the ML tree.

All the species newly analysed for this study (*Ch. regab, L. chuni*, *C. valdiviae*, *E. guiness*, *W. guineensis*, *I. perplexum*, *L. myriamae*, *C. n sp.I* and *C.n sp. II*), identified by expert taxonomists (R. Von Cosel and K. Olu), did actually group with their conspecifics from GenBank. *C. valdiviae* grouped with the species complex *C. pacifica*/*V. lepta*. The species identified as *L. chuni* was included within a clade of several species from the Japan Trench (BP = 92%). A relationship between an unidentified species from the Middle America Trench (*C. n. sp I*) and *E. extenta* (AF114388) is depicted in the ML tree. Two other unidentified specimens, one from the same area (*C. n sp CII*) and one from the Costa Rica accretionary wedge (AF114390) grouped together (Figure 2) with high bootstrap support (100%).

The molecular taxonomical reconstruction reported here provided limited support even for the recent taxonomic revision of vesicomyids based on morphological criteria. Upon 14 morphologically determined genera present in the data set only three were recovered as monophyletic: *Christineconcha*, *Abyssogena* and *Pliocardia*. The four haplotypes belonging to the genus *Christineconcha* clustered together with a maximum divergence of 0.6%, supporting the occurrence of a single species. Species belonging to the newly described genus *Abyssogena* [23] were monophyletic as well as species belonging to the genus *Pliocardia*.

The monophyly of the genus *Calyptogena* defined according to morphological criteria is broken due to the grouping of *C. fausta* with *V. lepta*. This clustering is quite unexpected but was also identified by Kojima *et al.* [16] as one of the pairs of trans-pacific species supported by bootstrap values greater than 90% (here 95%, Figure 2). This species may nevertheless deserve to be re-examined together with *V. lepta* specimens during the on-going genera revision. Species provisionally assigned to other genera by Krylova and Sahling [24] are not included in this cluster (e.g. *C. magnifica*). Similarly, one of the species provisionally assigned to the genus *Phreagena* (*C. nankaiensis*) is not included in the cluster gathering the other three species of this genus, *C. soyoae*, *C. kilmeri* and *C. okutani* [16].

Despite recent revisions [20], [21], [24], the reconstructed phylogeny presented here did not correspond to the current morphology-based taxonomy for the remaining genera, including *Wareniconcha*, *Laubiericoncha* and *Isorropodon*. Indeed, the genus *Wareniconcha* whose type species is *W. guineensis* from the Gulf of Guinea did not form a monophyletic cluster with the other provisionally assigned species *V. lepta*. Similarly, species of the genus *Phreagena* did not group together. Moreover, both species *L. myriamae* and *L. chuni* of the recently described genus *Laubiericoncha* do not form a monophyletic group, and *I. perplexum* and *C. fossajaponica* belonged to *Isorropodon*. Nevertheless these tree genera are represented by only two species in this study and additional taxa would be needed to confirm this observation.

In contrast, this phylogenetic reconstruction revealed well-supported groups that have yet to be defined, but confirmed some suggestions from previous studies. Indeed, Kojima *et al.* [16] identified 6 pairs of closely related (90 to 100% of similarity) trans-pacific species that were also observed here (e.g. *C. soyoae* and *C. kilmeri*, *V. magnocultellus* and *V. gigas*). The newly sequenced *L. chuni* from West Africa is closely related (divergence <2%) to an unidentified species sampled off Sanriku, Japan (BP = 100%) that suggest also Atlantic-Pacific similarities. Morphological identification of this unidentified specimen and phylogeographic analysis with more samples from both geographic areas would help determining whether these specimens belong to a single species exhibiting recent or contemporary exchanges, or sub-species with on-going divergence and lineage sorting. Moreover, both specimens were also close to *C. laubieri* and *V. gigas* from western and eastern Pacific Ocean, although they do not belong to the morphologically defined genera thus-far identified in this part of the world. Nevertheless, *L. chuni* and *C. laubieri* share many characters according to current study by Krylova (pers.com.) Further study on the systematic status of these specimens is needed. Other studied species were not related with well-supported nodes to previously known specimens. Barcoding and phylogenetic positioning of other species, particularly those from the Atlantic Ocean, may be necessary for a better understanding of the relationships among species.

As pointed out by Kojima et al. (2006) [19], *C. solidissima* and *C. kawamurai* appear as closely related and the species identified as *C. soldissima* is probably a junior synonym of *C. kawamurai*. Moreover, *C. fossajaponica* and both unidentified species from the Kurile Trench reported by Kojima et al. 2004 [16] and Okutani et al. 2009 [37] were very closely related. These three specimens may be conspecific as previously suggested by Okutani et al. 2009 [37].

Two cryptic species complexes have been highlighted by previous phylogenetic studies [12], [38]. The first complex corresponds to the species *V. lepta* and *C. pacifica* phylogenetically analysed by Peek *et al.* [15] who attributed the divergence to their respective distribution in vents versus seeps. In a later analysis including other lineages, Goffredi *et al.* [13] identified three more lineages within the same complex and suggested their bathymetric segregation. The divergence of the five lineages they identified in total is confirmed in the present study, with an obvious cluster gathering *C. pacifica* specimens (together with *C. storobogatovi* from hydrothermal vents) and another cluster of 4 lineages (*V. lepta*, mtII, mtIII, mtV) supported by high bootstrap values (Figure 2). Additionally, the species *C. fausta* also clustered in this group with the mtII lineage as its closest relative. *C. kilmeri*, initially included in the *C. kilmeri/V. gigas* complex of species, is clearly separated from *V. gigas* but undistinguishable from its Western Pacific counterpart *C. soyoae*, and closely related to another Japanese margin species (*C. okutanii*). In contrast, *V. gigas* are spread throughout three paraphyletic lineages supported by high bootstrap values, some also gathering Western Pacific sister species (*C. laubieri, V. magnocultellus*) and the new Western Africa *Laubiericoncha chuni*.(Figure 2).

To clarify the taxonomy of the two “new” species cryptic complexes *“V” gigas* and *“V.” lepta*, further morphologic and genetic studies are needed.

Both genera *Archivesica* and *Laubiericoncha* have gills with two pairs of demibranchs, a character also shared with *“C.”kilmeri* and *E. extenta* (in fact the whole cluster was supported by a 99% bootstrap value, except *C. okutani*, *C. kawamurai* and *C. soldissima*). On the contrary, the genus *Calyptogena*, the “*V”. lepta* complex, and the genera *Abyssogena*, *Christineconcha* and *Isorropodon* have a single pair of demibranchs. The presence of two demibranchs is considered as an ancestral character [20]. Besides this group of species, the species*“C.”magnifica* (new genus [24]) and *“C” packardana* branching in distinct clusters also shared this putative ancestral character. Ambiguous generic diagnoses may result from morphological convergences. The common occurrence of reduction in the hinge structures that would have evolved independently several times in the family, have been suggested to explain the lack of monophyly of generic names [24]. Soft part characters, such as specialised gill related to symbiosis (reduced outer gill demibranch, presence of wide interlamella septa), absence of pallial sinus or number of pallial apertures, may deserve careful consideration when describing new genera/species and it would also likely be interesting to compare their distribution with molecular phylogeny.

The discrepancies observed here between the most recent morphological taxonomy and our reconstructed molecular phylogeny highlights the necessity to carry on a re-evaluation of the morphological characters used in the identification of vesicomyid bivalves by performing as much as possible a systematic parallel morphological and molecular characterization. Furthermore, the molecular characterization of species assigned to some genera such as *Vesicomya, Isorropodon* and *Callogonia*, although not yet available, would increase the resolution of the phylogenetic tree.

### Phylogenetic perspective

The family Vesicomyidae constitutes a monophyletic group of relatively recent origin (50–80 million years) [12], [39]. However, results obtained here support a scenario of multiple migrations between eastern and western Pacific Ocean, as suggested by Kojima et al. (2004) [16]. Accordingly, several species, such as *V. sp mtII* and *C. fausta* and *C. kilmeri* and *C. soyoae*, cluster together despite their distinct distributional ranges across the Pacific Ocean.

The currently known biogeographic provinces and pathways, such as the Atlantic Equatorial Belt, that have been identified and are shared among various chemosynthetic taxa [40], [41], [42], [43], do not seem to apply to the set of vesicomyid species analysed in this study. Several species or species complexes of *Bathymodiolus* bivalves, gastropods and crustaceans exhibit an amphi-Atlantic distribution [40], whereas the vesicomyids collected from the eastern to western Atlantic (Gulf of Mexico, Barbados, Mid-Atlantic Ridge, Gulf of Guinea) are not closely related. Instead, they are scattered across the phylogenetic tree, except one specimen from the West Florida Escarpment (AF008280) that resolved as the sister lineage of a clade including *A. southwardae* from the Barbados Accretionary Prism and *V. sp* from the Mid-Atlantic Ridge. Further, none of the five vesicomyids sampled in the Gulf of Guinea across a large depth range (700–4000 m) seem to be related to any Atlantic seep or vent counterparts. This may be the result of either incomplete sampling or limited exchanges between the western and eastern parts of the Atlantic. Moreover, other species collected from the Gulf of Guinea did not form a monophyletic group either, although some of them occur in sympatry. For instance, *Ch. regab* and *L. chuni* were sampled on the Regab pockmark in the same species aggregate but are quite distant in the phylogenetic tree. Similarly, *C. valdiviae* and *E. guiness* occur on the same pockmark in the Guiness area, but were also distantly related in both phylogenetic and morphological [10] analyses. *W. guineensis* did not cluster with other species from the Gulf of Guinea. The lack of clustering of these sympatric species is similar to patterns observed for species that inhabit distinct geographic regions and oceans. This result suggests the occurrence of multiple series of speciation events followed by large-scale dispersal.

The phylogenetic reconstruction performed in the present study supports the findings of previous studies, suggesting that the speciation of the *C. pacifica/V. lepta* complex seems to be both related to the habitat (seep/vent) [15] and depth [13]. So far, *C. pacifica* seems restricted to seeps whereas *V. lepta* (mtIV) restricted to vents. The recent sampling of this complex at both seeps and vents in the basin would complete this analysis (Arnaud-Haond unpubl.). On the contrary, the lineages mtIII and mtV were only sampled at seeps while mtII is distributed in both ecosystems from Monterey Bay to Juan de Fuca along a relatively large depth range [13].

Interestingly both complexes of apparently cryptic species *V.lepta* and *V. gigas* have a wide bathymetric range and likely diverged in several lineages submitted to distinct environmental conditions. Both species complexes occur both at seeps and vents. In contrast *C. pacifica* and *C. kilmeri* have narrow bathymetric ranges and have been observed only in seeps thus far [20]).

### Preliminary attempt of character tracing

**Figure 3:**
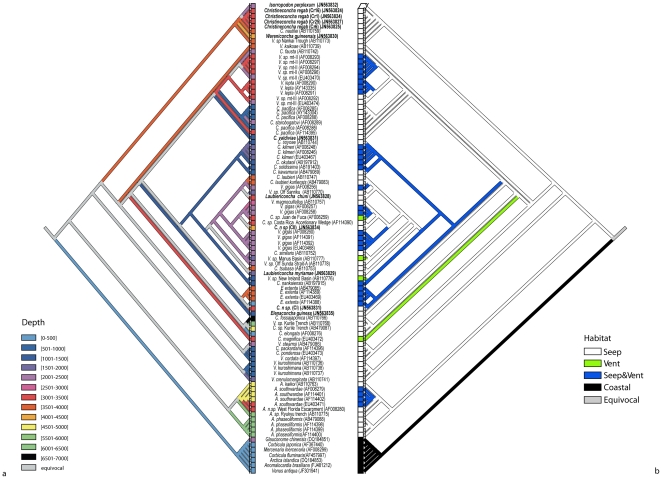
1-Ancestral character state reconstruction for the evolution of depth limits (a) and habitat (b) in bivalves based on unweighted parsimony was mapped onto the ML phylogeny. Character states are represented by colour-coded depth intervals. Branches with equivocal character states are shown in grey.

The influence of environmental parameters on the diversification and speciation in Vasicomyids was supported by the ancestral character reconstruction using the habitat and depth ranges of vesicomyid species (Figure 3). Both analyses showed a significant correlation between patterns of diversification and habitat/maximum depth where the species occur regardless the lack of resolution of some of the basal branches.

Vesicomyid bivalves exhibit a worldwide distribution and have colonized different chemosynthetic environments: hydrothermal vents, cold seeps, and whale falls [2], [3], [4]. The results reported here suggest that other factors rather than geographic boundaries have contributed to shape speciation patterns in vesicomyids. Recurrent events of ‘stepwise speciation’ seem to have occurred from shallow to deep waters in different ocean basins [13],[44]. Ancestral character state reconstructions performed here support this general trend (Fig. 2). In a recent review, Krylova and Sahling [24], reported the geographic and vertical distributions of vesicomyid genera and the species of the Gulf of Guinea seem to be depth-segregated [10]. Most genera occur in a single bathymetric zone; some have a bathyal (e.g. *Callogonia, Ectenagena, Elenaconcha*, etc.) distribution, whereas others are restricted to the abyssal zone (*Abyssogena* and *Christineconcha*) [24]. The deep Abyssogena genus clusters in the ML phylogenetic tree with *V. kuroshimana and V. crenulomarginata* (Figure 3), which are shallower species. Other species such as *E. guiness*, *C. elongata* and *C. kawamurai* show a more basal position in the phylogenetic tree although assigned to genera with upper bathyal distributions. These results would be in agreement with a putative pattern of diversification from the upper bathyal to abyssal depths. In other chemiosynthetic bivalve families such as Bathymodiolinae, phylogenetic analyses indicate that a seep species ancestor “gave rise to deep-sea hydrothermal vent mytilids” in an evolutionary progression from shallow- to deep-water habitats [45]. More recently, the “wooden steps to deep-sea vent” hypothesis has been supported by the basal position of sunken-wood species in the phylogeny [46], [47]. None of the whale-fall specimens (e.g., *V. gigas*) studied here and also inhabiting seeps and vents, showed a basal position in the phylogenetic tree but instead clustered with other conspecific specimens (Table S1). *W. guineensis* sampled in the vicinity of the Congo River channel could be associated to terrestrial organic matter accumulations. However, only vesicomyids larvae have been collected to date on sunken wood, during an in situ colonisation experiment with wood cubes deployed in the Nile deep-sea fan [48]. The absence of vesicomyid adults on sunken wood may either reflect their real status, incomplete sampling or extinction of the shallower species. The “wooden step” hypothesis in vesicomyids remains therefore an open question. The low resolution of the deepest nodes would render any conclusive interpretation of this character analysis highly speculative. The trend observed here on more recent and better-supported nodes opens however interesting perspectives. Also, adding further markers to the analysis could improve the resolution of the phylogeny and our understanding about the origin and evolutionary history of those widely distributed chemosynthetic taxa.

## Supporting Information

Table S1 **GenBank accession numbers, specimen collection sites and depth, maximum collection depth of species used for phylogeny reconstruction.** Specimen names are those of Genbank and new names are those from recent revisions of genera and examinations of specimen of ^1^Krylova *et al.* [24], ^2^Krylova and Sahling [20], ^3^Kojima *et al.* [16], ^4^Peek *et al.* [15], ^5^Okutani *et al.* [37] ^6^Goffredi *et al.* [13].
(DOCX)

## References

[1] McArthur AG, Tunnicliffe V, Mills RA, Harrison K (1998). Relics and antiquity revisited in the modern vent fauna.. Modern Ocean Floor Processes and the Geological Record.

[2] Sibuet M, Olu K (1998). Biogeography, biodiversity and fluid dependence of deep-sea cold-seep communities at active and passive margins.. Deep-Sea Research II.

[3] Tunnicliffe V, Barnes M (1991). The biology of hydrothermal vents: ecology and evolution.. Oceanogr Mar Biol Annu Rev: Aberdeen University Press.

[4] Bennett BA, Smith CR, Glaser B, Maybaum HL (1994). Faunal community structure of a chemoautotrophic assemblage on whale bones in the deep northeast Pacific ocean.. Marine Ecology Progress Series.

[5] Cavanaugh CM (1983). Symbiotic chemoautotrophic bacteria in marine invertebrates from sulphide-rich habitats.. Nature.

[6] Fiala-Médioni A, Le Pennec M (1988). Structural adaptation in the gill of the Japanese subduction zone bivalves (Vesicomyidae) *Calyptogena phaseoliformis* and *Calyptogena laubieri*.. Oceanologica Acta.

[7] Fisher CR (1990). Chemoautotrophic and methanotrophic symbioses in marine invertebrates.. Reviews in Aquatic Sciences.

[8] Fiala-Médioni A, Felbeck H (1990). Autotrophic processes in invertebrate nutrition: bacterial symbiosis in bivalve molluscs..

[9] Cosel Rv, Olu K (2008). A new genus and new species of Vesicomyidae (Mollusca: Bivalvia) from cold seeps on the Barbados accretionary prism, with comments on other species..

[10] Cosel Rv, Olu K (2009). Large Vesicomyidae (Mollusca: Bivalvia) from cold seeps in the Gulf of Guinea off the coasts of Gabon, Congo and northern Angola.. Deep Sea Research II.

[11] Vrijenhoek RC, Schutz SJ, Gustafson RG, Lutz RA (1994). Cryptic species of deep-sea clams (Mollusca: Bivalvia: vesicomyidae) from hydrothermal vent and cold-water seep environnements.. Deep-Sea Research.

[12] Peek AS, Gustafson RG (1997). Evolutionary relationships of deep-sea hydrothermal vent and cold-water seep clams (Bivalvia: Vesicomyidae): results from the mitochondrial cytochrome oxidase subunit I. Marine Biology.

[13] Goffredi SK, Hurtado LA, Hallam S, Vrijenhoek RC (2003). Evolutionary relationships of deep-sea vent and cold seep clams (Mollusca: Vesicomyidae) of the “*pacifica/lepta*” species complex.. Marine Biology.

[14] Peek AS, Feldman RA, Lutz RA, Vrijenhoek RC (1998). Cospeciation of chemoautotrophic bacteria and deep sea clams.. Proc Natl Acad Sci USA.

[15] Peek AS, Gaut BS, Feldman RA, Barry JP, Kochevar RE (2000). Neutral and nonneutral mitochondrial genetic variation in deep-sea clams from the family Vesicomyidae.. Journal of Molecular Evolution.

[16] Kojima S, Fujikura K, Okutani T (2004). Multiple trans-Pacific migrations of deep-sea vent:seep-endemic bivalves in the family Vesicomyidae.. Molecular Phylogenetics and Evolution.

[17] Kojima S, Kobayashi T, Hashimoto J, Ohta S (1995). RFLP analysis of a Mitochondrial gene, cytochrome oxidase I (COI) of three species of the genus *Calyptogena* around Japan.. Journal of Oceanography.

[18] Kojima S, Segawa R, Ohta S (1995). Molecular evidence that *Calyptogena laubieri* (Bivalvia: Vesicomyidae) is a valid sepcies.. Venus: Japanese Journal of Malacology.

[19] Kojima S, Tsuchida E, Numanami H, Fujikura K, Okutani T (2006). Synonymy of Calyptogena soldissima with Calyptogena kawamurai (Bivalvia: Vesicomyidae) and its population structure reveales by mitochondrial DNA sequences.. Zoological science.

[20] Krylova EM, Sahling H (2006). Recent bivalve molluscs of the genus *Calyptogena* (Vesicomyidae).. Journal of Molluscan Studies.

[21] Cosel Rv, Salas C (2002). Vesicomyidae (Mollusca: Bivalvia) of the genera *Vesicomya, Waisiuconcha, Isorrpodon* and *Callogonia* in the eastern Atlantic and the Mediterranean.. Sarsia.

[22] Krylova EM, Janssen R (2006). Vesicomyidae from Edison Seaomunt (South West Pacific:Papua New Guinea: New Ireland fore-arc basin).. Arch Molluskenkunde.

[23] Krylova EM, Sahling H, Janssen R (2010). *Abyssogena*: a new genus of the family Vesicomyidae (Bivalvia) from deep water vents and seeps.. Journal of Molluscan Studies.

[24] Krylova EM, Sahling H (2010). Vesicomyidae (Bivalvia): current taxonomy and distribution.. PLoS ONE.

[25] Krylova EM, Cosel Rv (2011). A new genus of large Vesicomyidae (Mollusca, Bivalvia, Vesicomyidae, Pliocardiinae) from the Congo margin, with the first record of the subfamily Pliocardiinae in the Bay of Biscay (northeastern Atlantic).. Zoosystema.

[26] Thiele J, Jaeckel S (1931). Muschelnder Deutschen Tiefsee-Expedition.Wis- senschaftliche Ergebnisseder DeutschenT iefsee- Expeditionaufdem Dampfer “Valdivia”.

[27] Sturany R (1896). Zoologische Ergebnisse VII. Mollusken I (Prosobranchier und Opisthobranchier; Scaphopoden; Lamellibranchier) gesammelt von S.M. Schiff “Pola” 1890–18.. Denkschriften der Kaiserlichen Akademie der Wissenschaften, Mathematische-Naturwissenschaftlischen Classe 63: 1–36,.

[28] Bohrmann G, Cruise participants (2008). Report and preliminary results of R/V METEOR Cruise M70/3, Iraklion – Iraklion, 21 November–8 December, 2006..

[29] Folmer O, Black M, Hoeh W, Lutz R, Vrijenhoek RC (1994). DNA primers for amplification of mitochondrial cytochrome C oxidase subunit I from metazoan invertebrates.. Molecular Marine Biology and Biotechnology.

[30] Mikkelsen PM, Bieler R, Kappner I, Rawlings TA (2006). Phylogeny of Veneroidea (Mollusca: Bivalvia) based on morphology and molecules.. Zool J Linn Soc.

[31] Drummond AJ, Ashton B, Cheung M, Heled J, Kearse M (2009). Geneious website.. http://www.geneious.com.

[32] Akaike H, BNPaF CSAKI (1973). Information Theory and an Extension of the Maximum Likelihood Principle.. Second International Symposium on Information Theory.

[33] Posada D, Crandall KA (1998). MODELTEST: testing the model of DNA substitution.. Bioinformatics.

[34] Guindon S, Gascuel O (2003). A simple, fast and accurate algorithm to estimate larges phylogenies by maximum likelihood.. Systematic Biology.

[35] Rodriguez FJ, Oliver JL, Marin A, Medina JR (1990). The general stochastic model of nucleotide substitution.. Journal of Theorical Biology.

[36] Maddison WP, Maddison DR (2001). MacClade 4 version.

[37] Okutani T, Koshi-Ishi T, Sato T, Imai T, Kato C (2009). Vesicomyid Fauna in the Chishima (Kurile) Trench: Occurrences of a New Taxon and Calyptogena extenta Venus.

[38] Baco AR, Smith CR, Peek AS, Roderick GK, Vrijenhoek RC (1999). The phylogenetic relationships of whale-fall vesicomyid clams based on mitochondrial COI DNA sequences.. Marine Ecology Progress Series.

[39] Little CTS, Vrijenhoek RC (2003). Are hydrothermal vent animals living fossils?. Trends in Ecology and Evolution.

[40] Olu K, Cordes EE, Fisher CR, Brooks JM, Sibuet M (2010). Biogeography and Potential Exchanges Among the Atlantic Equatorial Belt Cold-Seep Faunas.. PLoS ONE.

[41] Olu-Le Roy K, Cosel Rv, Hourdez S, Carney SL, Jollivet D (2007). Amphi-Atlantic cold-seep Bathymodiolus species complexes across the equatorial belt.. Deep Sea Research Part I: Oceanographic Research Papers.

[42] Cordes EE, Carney SL, Hourdez S, Carney RS, Brooks JM (2007). Cold seeps of the deep Gulf of Mexico: Community structure and biogeographic comparisons to Atlantic equatorial belt seep communities.. Deep Sea Research Part I: Oceanographic Research Papers.

[43] Genio L, Johnson SB, Vrijenhoek RC, Cunha MR, Tyler P (2008). New record of “*Bathymodiolus” mauritanicus* Cosel 2002 from the Gulf of Cadiz (NE Atlantic) mud volcanoes.. Journal of Shellfish Research.

[44] Fujikura K, Kojima S, Fujiwara Y, Hashimoto J, Okutani T (2000). New distribution records of vesicomyid bivalves from deep-sea chemosynthetic-based communities in Japanese waters.. Venus.

[45] Craddock C, Hoeh WR, Gustafson RG, Lutz RA, Hashimoto J (1995). Evolutionary relationships among deep-sea mytilids (Bivalvia: Mytilidae) from hydrothermal vents and cold-water methane/sulfide seeps.. Marine Biology.

[46] Samadi S, Quéméré E, Lorion J, Tillier A, von Cosel R (2007). Molecular phylogeny in mytilids supports the wooden steps to deep-sea vents hypothesis.. Comptes Rendus Biologies.

[47] Distel DL, Baco AR, Chuang E, Morrill W, Cananaugh C (2000). Do mussel take wooden steps to deep-sea vents?. Nature.

[48] Gaudron SM, Pradillon F, Pailleret M, Duperron S, Le Bris N (2010). Colonization of organic substrates deployed in deep-sea reducing habitats by symbiotic species and associated fauna.. Marine Environmental Research.

