# Supplementary material for: Phylogeny and Diversification Patterns among Vesicomyid Bivalves
Source: PLoS One. 2012 Apr 12;7(4):e33359. doi: 10.1371/journal.pone.0033359 (PMC3325225; doi:10.1371/journal.pone.0033359)
Supplement: Table S1 — GenBank accession numbers, specimen collection sites and depth, maximum collection depth of species used for phylogeny reconstruction. Specimen names are those of Genbank and new names are those from recent revisions of genera and examinations of specimen of 1Krylova et al. [24], 2Krylova and Sahling [20], 3Kojima et al. [16], 4Peek et al. [15], 5Okutani et al. [37] 6Goffredi et al. [13]. (DOCX) [file pone.0033359.s001.docx]

Table S1:

| Specimen | New name | Accession number | Sample site | Sample habitat | Sample depth | Upper range sample depth | Study |
| --- | --- | --- | --- | --- | --- | --- | --- |
| *Christineconcha regab* (Cr1) |  | JN563824 | Regab pockmark, Gulf of Guinea | Seep | 3167 | 3200 | This study |
| *Christineconcha regab* (Cr6) |  | JN563825 | Regab pockmark | Seep | 3170 | 3200 | This study |
| *Christineconcha regab* (Cr15) |  | JN563826 | Regab pockmark | Seep | 3167 | 3200 | This study |
| *Christineconcha regab* (Cr25) |  | JN563827 | Regab pockmark | Seep | 3167 | 3200 | This study |
| *Calyptogena valdiviae* |  | JN563831 | Guiness pockmarks, Gulf of Guinea | Seep | 687 | 2500 | This study |
| *Calyptogena n. sp* (CI) |  | JN563833 | Middle America Trench off Mexico | Seep | 3400 | 3400 | This study |
| *Calyptogena n. sp* (CII) |  | JN563834 | Middle America Trench off Mexico | Seep | 4300 | 4300 | This study |
| *Elenaconcha guiness* |  | JN563835 | Guiness pockmarks | Seep | 695 | 1200 | This study |
| *Isorropodon perplexum* |  | JN563832 | Amsterdam mud volcano, Mediterranean Sea | Seep | 2025 | 2500 | This study |
| *Laubiericoncha chuni* |  | JN563828 | Regab pockmark | Seep | 3170 | 3200 | This study |
| *Laubiericoncha myriamae* |  | JN563829 | Barbados Accretionary Prism | Seep | 1949 | 1949 | This study |
| *Wareniconcha guineensis* |  | JN563830 | ZC, Gulf of Guinea | Presumably Seep | 4000 | 4017 | This study |
| *Calyptogena phaseoliformis* | *Abyssogena phaseoliformis*^1^ | AB479088 | Kurile Trench | Seep | 4819 | 6400 | Okutani *et al.* 2009 |
| *Calyptogena sp.* | *Abyssogena phaseoliformis*^1^ | AF114398 | Aleutian Trench 'Shumagin' site | Seep | 4947 | 6400 | Peek *et al.* 2000 |
| *Calyptogena sp.* | *Abyssogena phaseoliformis*^1^ | AF114399 | Aleutian Trench 'Edge' site | Seep | 3550 | 6400 | Peek *et al.* 2000 |
| *Calyptogena sp.* | *Abyssogena phaseoliformis*^1^ | AF114400 | Aleutian Trench 'Shumagin' site | Seep | 3550 | 6400 | Peek *et al.* 2000 |
| *Calyptogena sp.* | *Abyssogena southwardae*^1^ | AF114401 | Logatchev Mid-Atlantic Ridge | Vent | 3038 | 5000 | Peek *et al.* 2000 |
| *Calyptogena sp.* | *Abyssogena southwardae*^1^ | AF114402 | Logatchev Mid-Atlantic Ridge | Vent | 3038 | 5000 | Peek *et al.* 2000 |
| *Calyptogena sp.* | *Abyssogena southwardae*^1^ | AF008279 | Barbados Accretionary Prism | Seep | 5000 | 5000 | Peek *et al.* 1997 |
| *Calyptogena extenta* | *Ectenagena extenta*^2^ | AB479085 | Peruvian Upper Slope Scarp | Seep | 3512 | 3512 | Okutani *et al.* 2009 |
| *Calyptogena n. sp.* | *Ectenagena extenta*^3^ | AF114388 | Kurile Trench | Seep | 3002 | 3512 | Peek *et al.* 2000 |
| *Calyptogena n. sp.* | *Ectenagena extenta*^3^ | AF114389 | Gorda Ridge | Vent | 3271 | 3512 | Peek *et al.* 2000 |
| *Ectenagena extenta* |  | EU403469 | Monterey Canyon | Seep | 1464 | 3512 | Steewart *et al.* 2008 |
| *Calyptogena elongata* |  | AF008276 | Santa Barbara Channel | Seep | 500 | 500 | Peek *et al.* 1997 |
| *Calyptogena fausta* |  | AB110742 | Off Toi, Suruga Bay | Seep | 1500-2200 | 2200 | Kojima *et al.* 2004 |
| *Calyptogena fossajaponica* |  | AB110766 | Japan Trench | Seep | 5400-6400 | 6809 | Kojima *et al.* 2004 |
| *Calyptogena kaikoi* | *Abyssogena kaikoi*^1^ | AB110763 | Off Muroto Point, Nankai Trough | Seep | 4800 | 4800 | Kojima *et al.* 2004 |
| *Calyptogena kawamurai* |  | AB479089 | Suruga Bay, Nankai Trough, Okinawa Trough | Seep | 300-900 | 900 | Okutani *et al.* 2009 |
| *Calyptogena sp* | *Calyptogena kilmeri*^4^ | AF008246 | Oregon Subduction Zone | Seep | 765 | 1765 | Peek *et al.* 1997 |
| *Calyptogena sp* | *Calyptogena kilmeri*^4^ | AF008248 | Guaymas Transform Fault | Seep | 1765 | 1765 | Peek *et al.* 1997 |
| *Calyptogena kilmeri* |  | EU403467 | Monterey Canyon | Seep | 970 | 1765 | Steewart *et al.* 2008 |
| *Calyptogena laubieri* |  | AB110747 | Daisan (third) Tenryu Submarine Canyon, Nankai Trough | Seep | 3800 | 3800 | Kojima *et al.* 2004 |
| *Calyptogena laubieri ssp* | *Calyptogena laubieri kurilensis*^5^ | AB479083 | Kurile Trench | Seep | 3560 | 3560 | Okutani *et al.* 2009 |
| *Calyptogena magnifica* |  | EU403472 | East Pacific Rise | Vent | 2507 | 3100 | Steewart *et al.* 2008 |
| *Calyptogena magnocultellus* |  | AB110757 | Yukie Ridge, Nankai Trough | Seep | 1900-2200 | 2535 | Kojima *et al.* 2004 |
| *Calyptogena nankaiensis* |  | AB197915 | Hiroo Submarine Canyon, Japan | Seep | 1200 | 1200 | Kojima *et al.* 2004 |
| *Calyptogena nautilei* |  | AB110759 | Zenisu Ridge, Japan | Seep | 3300 | 3835 | Kojima *et al.* 2004 |
| *Calyptogena n.sp. Costa Rica* |  | AF114390 | Costa Rica accretionary wedge | Seep | 3096 | 3096 | Peek *et al.* 2000 |
| *Calyptogena n.sp. West Florida Escarpment* | *Abyssogena* n. sp^1^ | AF008280 | West Florida Escarpment | Seep | 3313 | 3313 | Peek *et al.* 1997 |
| *Calyptogena okutanii* |  | AB197912 | Sagami Bay | Seep | 800-1500 | 1500 | Kojima *et al.* 2005 |
| *Calyptogena sp.* | *Calyptogena pacifica^4^* | AF008285 | Oregon Subduction Zone | Seep | 765 | 1000 | Peek *et al.* 1997 |
| *Calyptogena sp.* | *Calyptogena pacifica^4^* | AF008286 | Oregon Subduction Zone | Seep | 765 | 1000 | Peek *et al.* 1997 |
| *Calyptogena sp.* | *Calyptogena pacifica^4^* | AF008288 | Oregon Subduction Zone | Seep | 765 | 1000 | Peek *et al.* 1997 |
| *Vesicomya pacifica* | *Calyptogena pacifica^2^* | AY143304 | Invert Cliff, Monterey Bay | Seep | 1000 | 1000 | Goffredi *et al.* 2003 |
|  |  |  |  |  |  |  |  |
|  |  |  |  |  |  |  |  |
| *Calyptogena sp.* | *Calyptogena starobogatovi ^2^* | AF008289 | Oregon Subduction Zone | Seep | 2089 | 3096 | Peek *et al.* 1997 |
| *Calyptogena packardana* |  | AF114396 | Monterey Canyon | Seep | 635 | 635 | Peek *et al.* 2000 |
| *Calyptogena ponderosa* |  | EU403473 | Gulf of Mexico | Seep | 720 | 1097 | Steewart *et al.* 2008 |
| *Calyptogena similaris* |  | AB110752 | Tokai Thrust, Nankai Trough | Seep | 2100 | 2200 | Kojima *et al.* 2004 |
| *Calyptogena solidissima* |  | AB191403 | Minami-Ensei Knol, Okinawa Trough | Vent | 670-710 | 1018 | Kojima *et al.* 2006 |
| *Calyptogena sp. Japon Kurile Trench* |  | AB479087 | Kurile Trench | Seep | 4819 | 4819 | Okutani *et al.* 2009 |
| *Calyptogena soyoae* |  | AB110744 | Sagami Knoll, Sagami Bay | Seep | 1400-1500 | 1500 | Kojima *et al.* 2004 |
| *Calyptogena tsubasa* |  | AB110753 | Daisan (third) Tenryu Submarine Canyon, Nankai Trough | Seep | 3800 | 3800 | Kojima *et al.* 2004 |
| *Vesicomya cordata* |  | AF114397 | Green Canyon, Gulf of Mexico | Seep | 700 | 700 | Peek *et al.* 2000 |
| *Vesicomya crenulomarginata* |  | AB110741 | Dairoku (sixth) Kumano Knoll, Nankai Trough | Seep | 2000 | 2000 | Kojima *et al.* 2004 |
| *Calyptogena sp.* | *Vesicomya gigas^4^* | AF008256 | Juan de Fuca | Vent | 2416 | 2416 | Peek *et al.* 1997 |
| *Calyptogena sp.* | *Vesicomya gigas^4^* | AF008257 | Juan de Fuca | Vent | 2416 | 2416 | Peek *et al.* 1997 |
| *Calyptogena sp.* | *Vesicomya gigas^4^* | AF008258 | Middle Valley, Juan de Fuca Ridge | Vent | 2400 | 2416 | Peek *et al.* 1997 |
| *Vesicomya gigas* |  | EU403468 | Mendocino Fracture Zone | Seep | 1694 | 2416 | Steewart *et al.* 2008 |
| *Calyptogena sp.* | *Vesicomya gigas^4^* | AF008260 | Oregon Subduction Zone | Seep | 2028 | 2416 | Peek *et al.* 1997 |
| *Vesicomya gigas* |  | AF114392 | Santa Catalina Basin | Whale carcass | 1240 | 2416 | Peek *et al.* 2000 |
| *Vesicomya gigas* |  | AF114391 | Santa Catalina Basin | Whale carcass | 1240 | 2416 | Peek *et al.* 2000 |
| *Vesicomya kaikoae* |  | AB110739 | Off Muroto Point, Nankai Trough | Seep | 3600-3800 | 36800 | Kojima *et al.* 2004 |
| *Vesicomya kuroshimana* |  | AB110736 | Kuroshima Knoll | Seep | 700-810 | 810 | Kojima *et al.* 2004 |
| *Vesicomya kuroshimana* |  | AB110737 | Kuroshima Knoll | Seep | 700-810 | 810 | Kojima *et al.* 2004 |
| *Vesicomya kuroshimana* |  | AB110738 | Kuroshima Knoll | Seep | 700-810 | 810 | Kojima *et al.* 2004 |
| *Calyptogena sp.* | *Vesicomya sp.* mt-II*^6^* | AF008296 | Middle Valley, Juan de Fuca Ridge | Vent | 3416 | 3416 | Peek *et al.* 1997 |
| *Calyptogena sp.* | *Vesicomya sp.* mt-II*^6^* | AF008297 | Middle Valley, Juan de Fuca Ridge | Vent | 3416 | 3416 | Peek *et al.* 1997 |
| *Calyptogena sp.* | *Vesicomya sp.* mt-III*^6^* | AF008292 | Peruvian Upper Slope Scarp | Seep | 2500 | 3416 | Peek *et al.* 1997 |
| *Vesicomya lepta* |  | AY143335 | Guaymas Basin | Vent | 2020 | 3416 | Goffredi *et al.* 2003 |
| *Calyptogena sp.* | *Vesicomya lepta^4^* | AF008290 | Guaymas Basin | Vent | 2020 | 3416 | Peek *et al.* 1997 |
| *Calyptogena sp.* | *Vesicomya lepta^6^* | AF008291 | Guaymas Basin | Vent | 2016 | 3416 | Peek *et al.* 1997 |
| *Vesicomya lepta* | *Vesicomya sp*. mt-V^6^ | AF114395 | Costa Rica accretionary wedge | Seep | 3096 | 3416 | Peek *et al.* 2000 |
| *Calyptogena sp.* | *Vesicomya sp.* mt-II*^6^* | AF008293 | Juan de Fuca | Vent | 2437 | 3416 | Peek *et al.* 1997 |
| *Calyptogena sp.* | *Vesicomya sp.* mt-II*^6^* | AF008294 | Juan de Fuca | Vent | 1547 | 3416 | Peek *et al.* 1997 |
| *Calyptogena sp.* Juan de Fuca |  | AF008259 | Middle Valley, Juan de Fuca Ridge | Vent | 2400 | 3416 | Peek *et al.* 1997 |
| *Vesicomya sp.* MAR | *Abyssogena southwardae*^1^ | EU403471 | Logatchev, Mid-Atlantic Ridge | Vent | 3028 | 3028 | Steewart *et al.* 2008 |
| *Vesicomya sp.* mt-II |  | EU403470 | Juan de Fuca | Vent | 2200 | 2200 | Steewart *et al.* 2008 |
| *Vesicomya sp.* mt-III |  | EU403474 | Monterey Bay | Seep | 2200 | 2200 | Steewart *et al.* 2008 |
| *Vesicomyidae sp.* 'Kurile Trench and Japan Trench |  | AB110768 | Japan Trench and Kurile Trench | Seep | 4700-6400 | 6400 | Kojima *et al.* 2004 |
| *Vesicomyidae sp.* 'Manus Basin' |  | AB110777 | DESMOS site, Manus Basin | Vent | 1900 | 1900 | Kojima *et al.* 2004 |
| *Vesicomyidae sp.* 'Nankai Trough' |  | AB110773 | Off Muroto Point, Nankai Trough |  | 3600-3800 | 3800 | Kojima *et al.* 2004 |
| *Vesicomyidae sp.* 'New Ireland Basin' |  | AB110776 | Edison Seamount, New Ireland Basin | Vent | 1450 | 1450 | Kojima *et al.* 2004 |
| *Vesicomyidae sp.* 'off Sanriku' |  | AB110770 | Off Sanriku | Seep | 1700 | 1700 | Kojima *et al.* 2004 |
| *Vesicomyidae sp*. 'off Sunda Strait-A' |  | AB110778 | Off Sunda Strait-A | Seep | 2100 | 2100 | Kojima *et al.* 2004 |
| *Vesicomyidae sp.* 'Ryukyu Trench' | *Abyssogena sp*^1^ | AB110775 | Ryukyu Trench | Seep | 5900 | 5900 | Kojima *et al.* 2004 |
| *Vesicomya stearnsii* |  | AB479086 | Gulf of California | Seep | 3070 | 2500 | Okutani *et al.* 2009 |
| *Glauconome chinensis* |  | DQ184851 | Western Pacific | Costal | 0 | 0 | Mikkelsen *et al.* 2006 |
| *Venus antiqua* |  | JF301941 |  | Costal |  | 100 | Pierto *et al.* 2011 |
| *Anomalocardia brasiliana* |  | FJ481212 | Brazil | Costal |  | 1.5 | Arruda *et al.* 2009 |
| *Mercenaria mercenaria* |  | AF008299 | Atlantic | Costal |  | 15 | Peek *et al.* 1997 |
| *Arctica islandica* |  | DQ184853 |  | Costal |  | 480 | Mikkelsen *et al.* 2006 |
| *Corbicula fluminaris* |  | AF457997 | Asian | Costal |  | 100 | Park *et al.* 2001 |
| *Corbicula japonica* |  | AF367440 | Korean | Costal |  | 5.4 | Park *et al.* 2001 |
